# Some Practical Problems in Sociology Shown by a Study of the Southern Negro

**Published:** 1902-10

**Authors:** M. L. Perry

**Affiliations:** Pathologist to the State Sanitarium, Milledgeville, Ga.


### SOME PRACTICAL PROBLEMS IN SOCIOLOGY SHOWN BY A STUDY OF THE SOUTHERN NEGRO.*

BY M. L. PERRY, M.D.,

Pathologist to the State Sanitarium, Milledgeville, Ga.

According to the census of 1900 the total population of the United States is something over seventy-six millions. Of this number there are 8,840,789 persons of negro descent, over ninety per cent. of whom are residents of the Southern States. To come closer home, there are in Georgia 1,034,813 negroes out of a total population of about two and one-fourth millions. These figures show the important rôle which the negro must necessarily play in our social system, especially here in the South, and to what extent society at large may be influenced by the degree of his development. That the writers and students of sociology throughout the country have been awake to the importance of this subject is shown by the amount of literature which has appeared within the last decade treating of the negro question. Much of this literature, however, has had either a political aspect or has dealt with the social status of the negro race as compared with the white. I do not propose to touch upon either of these subjects. It is the object of this paper to present for your consideration certain conditions existing in the Southern negro which have a profound influence upon their development as a race, and, thus, upon society at large. These conditions are well known to the medical profession, but the laity should, I think, have a better knowledge of them.

First, I will call your attention to the prevalence of venereal

* Read before the Georgia Sociological Society, Atlanta, June, 1902.

diseases. The negro is notoriously lax in his morals. There is in his make-up a large endowment of animal passion, while judgment and the proper consideration of consequences are usually deficient. Having these natural proclivities, let him be placed in an environment where restraint is slight and social caste does not depend upon virtue and chastity (and we know that this is the custom among the negroes in many sections), and what is the result? It can only lead to wide-spread and promiscuous sexual indulgence. Under such circumstances venereal diseases are always prevalent. That venereal diseases do exist to an alarming degree among the negroes in the cities and larger towns is a fact that can be attested by any physician. It is true also that this condition has developed in comparatively recent years; certainly since the days of slavery.

There are two diseases, viz.: gonorrhea and syphilis, which deserve some special attention. They deserve special consideration because both of these loathsome maladies may be, and are, at times, communicated to innocent individuals through the ignorance or carelessness of the person afflicted with them. Let a servant who, in the discharge of his or her duties, is thrown in daily contact with the members of a household, or a nurse who has the charge of innocent and defenceless children, be afflicted with either gonorrhea or syphilis, and there hangs constantly over that family a blight like the sword of Damocles, ready to fall at any time upon some unsuspecting member. I know of no more distressing sight than to see a pure and guileless child suffering with a loathsome venereal disease. And, yet, such cases now and again come to the knowledge of the physician. This is a question which must necessarily appeal to us here, in this section of the South, where we have to depend almost entirely upon the negro race for our domestic servants.

The sociological influence of gonorrhea is limited almost entirely to this question of its communication; but it is not so with syphilis. Here we have to deal with a disease which is insidious in its onset, obstinate in its course, persistent in its attacks upon its victim and whose influence, like the iniquities of which we read in Holy Writ, may be visited upon the children unto the third and fourth generation. Syphilis, at the present time, is making inroads upon the negroes of the South, and its influence will be far-reaching in its effects upon the race. This is due, primarily, to the fact that the negro rarely becomes cured of the disease. As a usual thing they apply for relief from certain disagreeable symptoms, and as soon as this is obtained they give up treatment. Some years since, while serving as interne in a hospital in a southern city, I had abundant opportunity to note this fact. I do not remember but one patient who continued treatment for any length of time after the annoying symptoms had disappeared. Thus, the negro who is afflicted with syphilis goes about for years, it may be, infecting others and procreating children weakened by inherited disease and unfit to assume their place in society.

Tuberculosis is another disease which has, in recent years, become so prevalent among the negroes as to justify the belief that it may soon be a veritable scourge to the race. The susceptibility of the negro to consumption has apparently undergone a most remarkable change within the last thirty or forty years. During the days of slavery the disease was so seldom found among them that they were considered almost immune to it. In fact, some of the older writers took that stand squarely, and asserted that consumption was unknown in the race. From being thus so rare as to be almost unknown, it has, in a single generation, become so prevalent and so fatal that now more negroes in the South are dying with tuberculosis than any other disease. While it exists among all classes, it is in the densely populated quarters ot towns and cities, where they live in overcrowded and poorly ventilated houses, that it is found to be most frequent. In institutions, where large numbers are more or less closely confined, the ravages of this disease are becoming truly alarming. In this connection, it may be of interest to state that of the cases coming to autopsy in the negro department of the Georgia State Sanitarium last year, 50 per cent. showed some tubercular involvement, and from observation and study of the subject for a period of three years in that institution, I am of the opinion that this per cent. may be considered a fair index of the existence of this disease in all our negro patients at the time of death. This high per cent. is of course, not applicable to the race in general, but it is a fact beyond question that tuberculosis exists wide-spread among the negroes of this section, and, at the present time, is on the increase.

From the consideration of this state of affairs two questions of great importance, from a sociological point of view, present themselves. They are: First, what is its influence upon society, and second, what will be the effect upon the race? When we reflect upon the fact that tuberculosis is infectious and, under favorable circumstances, may be communicated from one individual to another, and that its spread can be checked only by careful attention to personal hygiene, the answer to the first question must be apparent to all. We may safely say that nine-tenths of all the negroes afflicted with consumption are utterly ignorant of its communicability, and, also, of the hygienic precautions necessary for its restriction. The influence upon society of a constantly increasing number of individuals, each of whom is a source of infection of one of the most dreaded and fatal diseases known to mankind, must necessarily be baneful.

The effect upon the race of such wholesale infection must also be bad. It has long been recognized in medicine that the descendants of tuberculous parents are below the normal standard in their resistance to disease. As this taint becomes more pronounced in the race, their physical development will suffer. Indeed, such a change has apparently already begun. The older writers upon the subject emphasize the fact that prior to 1860 the negro was a splendid specimen physically. The following quotation from a paper on "The Future of the Colored Race in the United States," published some years since, by Dr. Eugene Corson, is interesting in this connection. He says: All the information which I have been able to obtain has satisfied me that the race was a healthy one, even healthier in the main than the whites. Since the war things have been reversed; the colored race as a race is not a healthy and robust one; their vitality is in a state of unstable equilibrium, liable from any undue strain to give way. Statistics from institutions, having both white and colored patients, will show that the latter are less able to resist the onslaught of disease. Dr. Mitchell, the superintendent of the Mississippi State Insane Hospital, in his last report says: There is one great difference between the races as regards mortality, and although our treatment, both dietetic and medicinal, is the same, our loss from deaths among the colored far exceeds that sustained by the whites.

Let us see what the records of the State Sanitarium of Georgia show. From the report for the year ending September 1st, 1900, we find that the death-rate, based upon the total number under treatment, was in the white department, 5.8 per cent., and in the colored department, 11.5 per cent. From the last report, being for the year ending September 1st, 1901, we find a death-rate among the whites of 6.7 per cent., while in the negro department it reached 17.1 per cent. This present year, the ratio up to this time is even greater, and this, despite the fact that their care and treatment is the same. These are facts, not theories. They show that under similar conditions the negro at the present time offers less resistance to disease than does the white man, and, therefore, is a less stable specimen physically. To just what extent syphilis and tuberculosis are responsible for this weakened vitality in the negro is, of course, impossible to say definitely. I believe, however, that the facts will warrant the assumption that their rôle has been a major one.

Turning from the physical condition of the negro, let us now investigate the mental stability of the race. Statistics, which are always more important and reliable than mere theories and opinions, show us here a most extraordinary state of affairs. They show us that since 1860 the negro race in this section (and the same is true of the South generally) has undergone such a change in their tendency toward the development of mental diseases as is shown by no other people in a similarly short period of time in the whole history of mankind. I will take the statistics of our own State, Georgia, a typical Southern State, as they will probably be of more interest to this Society than would those of the whole United States.

According to the census of 1860, there were in the State of Georgia 44 insane negroes. The negro population at that time was 465,698. The ratio of insane to the total population was thus 1–10584.

In 1870 there were 129 insane negroes in this State, out of a total colored population of 545,142, or 1–4225.

In 1880 the census shows that there were in the State 411 colored insane, out of a total of 725,133, or 1–1764.

In 1890, according to the census, there were in Georgia 560 insane negroes, out of a population of 858,815, or 1–1533.

The census of 1900 gives the negro population of Georgia as 1,034,813. The bulletin giving the insane population has not yet been issued, but we can approximate very closely to what it will be. There were in the State Sanitarium, September 1st, 1900, 799 colored patients, and from the number of applications for admission at that time, we may safely estimate that there were, at least, 100 more insane in the State. Reckoning on this basis of 900, we find the ratio of insane to the total colored population in 1900 to be 1-1149.

Thus in forty years the total negro population in the State has been a little more than doubled, while the number of insane has increased twentyfold. No other such rapid and radical change in the mental stability of a race is recorded in history. Can any one doubt but that the race has been weakened by it? This outburst of insanity becomes still more remarkable when we consider that for generations prior to 1860 the colored people had been free from mental disease. It has developed, therefore, without the slightest hereditary taint. What their condition will be at the end of the next forty years can only be conjectured, but considering the fact that the rising generation will have to contend with a marked hereditary predisposition from which their fathers were free, the outlook in this direction is certainly not encouraging. Even now the brain of the negro does not appear to be able to sustain a mental strain as well as that of a white man. This is shown by the fact, well recognized in asylum practice, that the colored insane tend to pass through the primary insanities and become demented more quickly than do the whites. A word or two upon the comparative brain capacity of the two races may be of interest here. I know of no statistics giving comparative brain-weights in the sane, but I have taken for comparison the brains of 100 patients of each race dying insane.

The following table gives the average brain-weight in these cases, together with the average normal weight:

Average weight of normal brain in human male, 1403 gms., female, 1247 gms.

Average brain-weight in 100 white patients, dying insane, male, 1353 gms., female, 1197 gms.

Average brain-weight in 100 colored patients, dying insane, male 1229 gms., female, 1092 gms.

The average weight of the brain of the negro is thus seen to fall below that of the white man by something more than 100 gms., or about 3½ oz. It is true that the degree of intelligence is indicated more by the development than by the weight of the brain, but other things being equal, the average brain-weight of a race may be taken as an index of its mental capacity.

From the foregoing we see that within the past few decades there has been a decided tendency toward the lowering of the status of the colored race, both as regards physical condition and mental stability. Let us see if we can correlate these conditions and find what has been the cause of this radical alteration in the racial temperament. We have seen that they were remarkably free from that most important of all predisposing factors in the production of disease, viz.: hereditary taint. They have been by nature likewise freed from cares and the harassing burdens of responsibility incident to business and public life. The mixing of the races has by some been offered as a probable factor in the weakening of the colored race. This is untenable, however, for among the insane, where such effect would be most likely to show, we find the full bloods far outnumbering the mulattoes. There is only one influence to which we can attribute their altered condition ; that is, environment, which is applicable to both the physical and mental temperament. The sudden removal of those healthy restraints which for generations past had surrounded them during the days of slavery was followed by the taking-up of vicious habits of indulgence. The tendency to flock to the cities and towns, which has been and still is so pronounced with the negroes, has had a deleterious effect upon them. There they readily fall into idle habits which are the breeders of vice and crime. It is in these overcrowded districts that syphilis and tuberculosis are found in greatest profusion. The laws of health and hygiene, which in the olden days were strictly enforced, are now too often quite neglected. Unless something can be done either by education or legal enactment to improve this unwholesome and pernicious environment, which to-day surrounds a large element of the negro race in the South, I can only believe that they will go on from bad to worse.

I cannot leave this subject without calling attention to one more danger which is threatening the negro at this time: that is the drug habit. We are all of us more or less familiar with its bad effect upon the whites, but, believe me, if the colored people, with their strong natural bent toward the indulgence of the appetites, become the victim of drugs and yield to the subtile and delusive enchantment of opium and cocain the result will be disastrous. There is nothing which has such a benumbing and perverting action upon the moral nature of an individual, to say nothing of the physical and mental effect, as the chronic poisoning by the use of these drugs. I am reliably informed that in some sections of the South their habitual use is already becoming prevalent. So far as I can learn, it has made little headway as yet in Georgia, but a start has been made. Here in Atlanta I am informed by good authority, that, until a few months ago, the habit was decidedly upon the increase, and it had reached such proportions among the lower classes, to whom it seemed to be chiefly limited, that 75 per cent. of the women and 20 per cent. of the men coming to the station-house were addicted to the use of cocain. Since the passage of a law prohibiting the sale of cocain, except upon prescription, the habit appears to be decreasing. This suggests the remedy, and it is our bounden duty, as the governing and controlling race, to protect these people by the enactment and strict enforcement of such laws in every State.

In conclusion, let me say that I do not wish to appear as an alarmist nor a confirmed pessimist. I have merely presented some facts as they exist. If they are such as to warrant a pessimistic view upon the subject, then the quicker we realize it the better, that we may look about us for some remedy to apply.

Nor do I wish this paper to in any way be misconstrued into an attack upon the southern negro. Far be it from me to cast any reflection upon those honest and honorable negroes who are living right and endeavoring to raise their race to a higher level. It is the erring and afflicted to whom this paper refers, and it has been written only in the hope that through a wider knowledge of the facts some good might come to the negro race and to society at large.

